# Dynamics of Whole Virus and Non-Structural Protein 1 (NS1) IgG Response in Mice Immunized with Two Commercial Tick-Borne Encephalitis Vaccines

**DOI:** 10.3390/vaccines10071001

**Published:** 2022-06-23

**Authors:** Jiri Salat, Petra Strakova, Daniel Ruzek

**Affiliations:** 1 Laboratory of Emerging Viral Infections, Veterinary Research Institute, Hudcova 70, CZ-62100 Brno, Czech Republic; petra.strakova@vri.cz; 2 Laboratory of Arbovirology, Institute of Parasitology, Biology Centre of the Czech Academy of Sciences, Branisovska 31, CZ-37005 Ceske Budejovice, Czech Republic; 3 Department of Experimental Biology, Faculty of Science, Masaryk University, Kamenice 735/5, CZ-62500 Brno, Czech Republic

**Keywords:** tick-borne encephalitis virus, vaccine, non-structural protein 1

## Abstract

The presence of a non-structural protein 1 (NS1) in tick-borne encephalitis (TBE) vaccines and the possible induction of an NS1-specific immune response in vaccinated individuals remains a somewhat controversial topic. Previously, we detected the presence of NS1 in the Encepur TBE vaccine by mass spectrometry and found the induction of NS1-specific IgG antibodies in mice vaccinated with the FSME-Immun TBE vaccine. Here, in this follow-up study, we examined the dynamics and extent of the NS1-specific IgG response in mice vaccinated with these two vaccines in more detail and compared it with the IgG response to the whole virus (WV). Mice were vaccinated at two-week intervals with a total of six doses of each vaccine, and levels of IgG antibodies to TBE virus WV and NS1 were measured by ELISA after each dose. Both vaccines elicited a robust anti-WV IgG response after two doses. The Encepur vaccine did not elicit NS1-specific IgG even after all six doses. In contrast, the FSME-Immun vaccine triggered the production of NS1-specific IgG after four doses. The results indicate that FSME-Immun is the only vaccine that elicits an NS1-specific antibody response in mice. However, compared to WV-specific IgG, the NS1-specific response is weaker, and a higher number of doses is required to induce detectable levels of NS1-specific IgG antibodies.

## 1. Introduction

Tick-borne encephalitis (TBE) is a tick-borne zoonosis caused by the TBE virus (TBEV), a member of the genus *Flavivirus*, family *Flaviviridae* [1]. TBEV is endemic in much of Europe and Asia [2], and recently the virus has been detected also in northern Africa [3]. TBE can manifest as a mild flu-like illness or progress to neurological disease, typically manifesting as meningitis, meningoencephalitis, or encephalomyelitis [2,4]. TBE diagnosis relies primarily on serologic testing to detect whole virus (WV)-specific IgM and IgG antibodies [2,5,6]. Because the TBE vaccines currently in use are based on purified and inactivated TBEV particles [2], detection of WV-specific antibodies does not allow differentiation between antibodies elicited after vaccination and infection. Previously, it was assumed that the NS1 antigen is not present in current TBE vaccines and that those vaccinated, therefore, do not develop an NS1-specific antibody response. Therefore, serology based on the detection of antibodies to NS1 is believed to be a promising tool to distinguish between TBE antibodies induced by infection and those induced by vaccination [7]. However, our recent study showed that two commercial TBE vaccines do contain NS1 antigens [8]. This was demonstrated by a combination of experimental approaches that included mass spectrometry analysis of the vaccine content and analysis of NS1-specific antibody response in mice immunized with these vaccines. Mass spectrometry analysis of the Encepur vaccine provided clear evidence for the presence of both the envelope (E) protein (as the major surface antigen of the WV) and NS1 antigens in the vaccine preparation. Interestingly, despite the presence of detectable levels of NS1 antigens in the vaccine preparation, the Encepur vaccine did not elicit an NS1-specific serological response in mice even after six doses were administered [8]. Mass spectrometric analysis of the vaccine FSME-Immun failed in part because of the high content of human serum albumin used as a stabilizer in the vaccine (sucrose is used as a stabilizer in the Encepur vaccine) [8]. Thus, only the E antigen, and not NS1, was identified in this vaccine. However, the presence of the NS1 antigen in FSME-Immun was confirmed by vaccination of naïve mice, which elicited a strong anti-NS1 antibody response in addition to anti-WV antibodies [8]. 

Whether immunization with TBE vaccines elicits an NS1-specific antibody response in humans remains difficult to say. In previous studies with vaccinated individuals (in most cases with ≤3 doses of the vaccine), NS1-specific antibodies were not detected in the majority of cases [7,9,10]. Consistent with these studies, our previous work in a small cohort of vaccinated individuals found that most vaccinees who received ≤3 doses were negative for NS1-specific antibodies. In some vaccinated individuals who had received more than three doses of the vaccine, anti-NS1 antibodies were detected in serum by NS1-specific Western blot analysis [8]. 

Therefore, the possible NS1-specific antibody response in vaccinated individuals remains a controversial topic. To obtain more data and remove possible doubts about our previous work, we decided to repeat our previous experiments with the vaccination of mice in this follow-up study with new lots of the two commercial TBE vaccines and to characterize in more detail the dynamics of the NS1-specific antibody response in the vaccinated animals. Our previous experiments were repeated (i) with a different vaccine batch to see if the NS1-specific antibody response was dependent on a particular vaccine batch, (ii) with more mice per group to increase the statistical power of the analysis, and (iii) with analysis of WV and NS1-specific IgG levels after each vaccine administration to characterize the detailed dynamics and extent of the immune response.

## 2. Materials and Methods

### 2.1. Ethics Statement

Animal experiments were performed in accordance with Czech laws and guidelines for the use of laboratory animals. The protocol was approved by the Departmental Expert Committee for the Approval of Projects of Experiments on Animals of the Ministry of Agriculture of the Czech Republic and the Committee on the Ethics of Animal Experimentation at the Veterinary Research Institute (Approval No. 26674/2020-MZE-18134).

### 2.2. Vaccination of Mice

BALB/c mice (females, 6 weeks old, Envigo) were used for the vaccination experiment. Mice in the first group (N = 6) were vaccinated with Encepur (GSK Vaccines, Brentford, UK; Lot No.: AEA33A1C). The second group of mice (N = 6) were vaccinated with FSME-Immun (Pfizer, New York, NY; Lot No.: EK3932). The mice in the third group (N = 6) received only the adjuvant and served as controls. A single vaccine dose consisted of a mixture of vaccine antigen (0.25 µg, corresponding to 52.8 µL of FSME-Immun, or 83.3 µL of Encepur), 10% Alhydrogel adjuvant (15 µL/dose; InvivoGen, San Diego, CA, USA), and PBS (Serana Europe GmbH, Pessin, Germany). The total volume of the vaccine dose was 0.15 mL. Vaccine doses were prepared immediately before use. Mice were vaccinated subcutaneously dorsally in the neck region. A total of six vaccine doses were administered at two-weeks intervals. Blood samples were collected from the tail vein of the mice 7 days after each vaccination. The concentration of specific anti-NS1 TBEV antibodies and anti-whole virus TBEV antibodies was measured by ELISA.

### 2.3. Detection of Antibodies in Mouse Sera

Detection of anti-NS1 specific IgG antibodies in the sera tested was performed using the Mouse Anti-Tick-Borne Encephalitis virus NS1 IgG Elisa Kit (Alpha Diagnostic International, San Antonio, TX, USA) according to the manufacturer’s instructions. The assay used evaluates the concentration of specific anti-NS1 antibodies in arbitrary units (U/mL). Anti-TBEV whole virus IgG antibodies in the tested sera were measured using the IMMUNOZYM FSME IgG All-Species Kit (Progen GmbH, Heidelberg, Germany) following the manufacturer’s instructions. The assay evaluates the concentration of specific IgG antibodies to TBEV in Vienna units (VIEU/mL).

### 2.4. Statistical Analysis

Differences in antibody levels between the tested groups were analyzed using Mann–Whitney *U* test. The analyses were performed by GraphPad Prism 7 for Windows (version 7.04). A *p*-value of <0.05 was considered significant.

## 3. Results and Discussion

The NS1-specific antibody response after vaccination with the commercial vaccines has been controversial because other studies have not found NS1-specific antibodies in serum samples from human vaccinees with completed vaccination regimens [7,9,10,11]. One recent study compared the dynamics and extent of NS1-antibody responses in TBE vaccination breakthroughs and unvaccinated TBE patients and found that neither the dynamics nor the extent of NS1-antibody formation differed significantly between these two groups, arguing against substantial NS1-specific priming and an anamnestic NS1-antibody response in vaccination breakthroughs [12]. The present study not only confirmed the previous results but also provided a more detailed insight into the kinetics and magnitude of the NS1-specific IgG antibody response in the vaccinated mice. 

**Figure 1:**
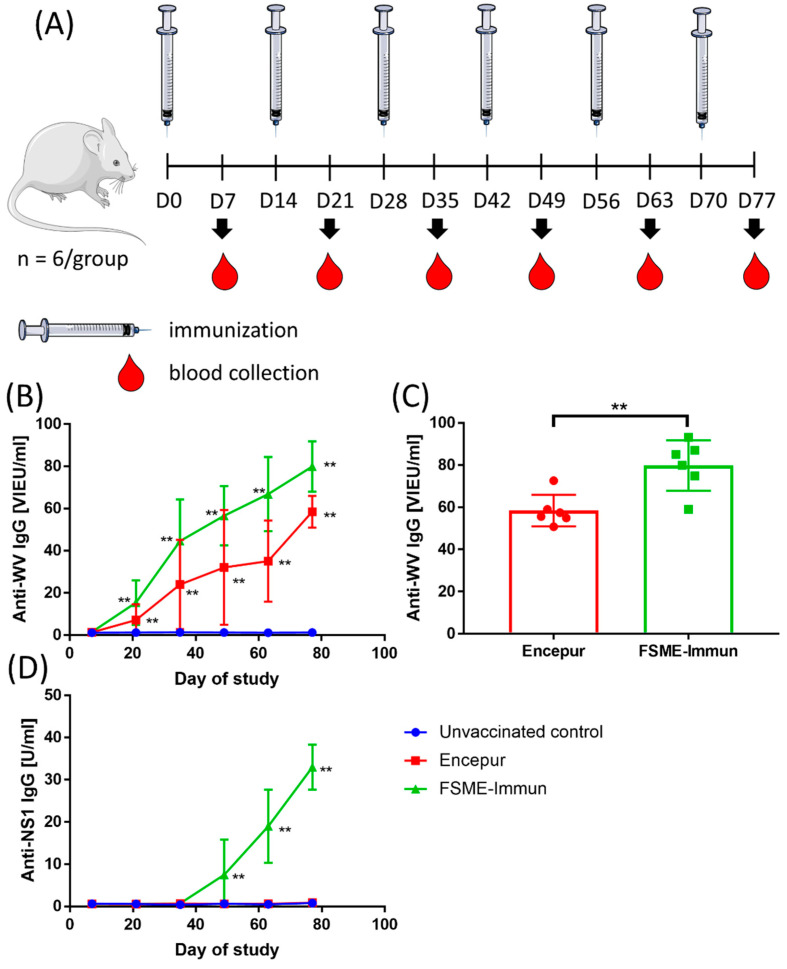
(**A**) Experimental protocol. Mice were immunized with either Encepur or FSME-Immun vaccines. Control mice received only the adjuvant. A total of six doses of vaccine were administered two weeks apart. Blood samples were collected from the tail vein of the mice 7 days after each vaccination. (Figure created using Servier Medical Art, available at www.servier.com, accessed on 1 April 2021). (**B**) Dynamics of whole virus (WV)-specific IgG in mice vaccinated with either Encepur or FSME-Immun. Mice receiving only the adjuvant were used as controls. Concentrations of anti-WV IgG in vaccinated mice were statistically compared with unvaccinated controls. (**C**) Comparison of WV-specific IgG levels in mice vaccinated with six doses of Encepur or FSME-Immun vaccine. (**D**) Dynamics of NS1-specific IgG in mice immunized with either Encepur or FSME-Immun vaccines. Mice receiving adjuvant only were used as controls. Concentrations of anti-NS1 IgG in vaccinated mice were statistically compared with unvaccinated controls. **, *p* < 0.01.

Similar to the previous study [8], mice received six doses of the vaccine 2 weeks apart. Negative controls received six doses of the adjuvant. However, unlike in the previous study, blood samples were collected seven days after each dose, and the concentrations of WV- and NS1-specific IgG were measured (Figure 1A). 

The kinetics of the WN-specific IgG antibody response was similar for both vaccines. Significantly increased (*p* < 0.01) concentrations of WN-specific IgG were detected after two doses of either vaccine, and then the concentrations increased with the number of vaccine doses administered (Figure 1B). At the end of the experiment, i.e., after six doses, significantly higher levels of WV-specific IgG (*p* < 0.01) were detected in mice immunized with the FSME-Immun vaccine than in mice receiving the Encepur vaccine (Figure 1C). 

Consistent with the results of the previous study [8], all blood samples from mice immunized with the Encepur vaccine were negative for the presence of NS1-specific IgG (Figure 1D). In contrast, mice immunized with FSME-Immun developed detectable levels (*p* < 0.01) of NS1-specific IgG after four doses, and the levels of these antibodies then increased after each subsequent dose until the end of the experiment (Figure 1D). Unfortunately, a comparison of NS1-specific IgG levels obtained in our previous and current study was not possible because different standards and arbitrary units were used in the previous and current ELISA assays, albeit from the same manufacturer. However, it appears that the NS1 IgG response is independent of the vaccine lot used, as the positive results in the case of FSME-Immun and the negative results in the case of Encepur were obtained analogously with different vaccine lots than those used in the previous study [8].

The results show that FSME-Immun is the only vaccine that elicits an NS1-specific antibody response in mice. Why the Encepur vaccine does not elicit an NS1-specific antibody response, despite containing detectable levels of NS1 antigen [8], remains enigmatic. After FSME-Immun vaccination, the NS1-specific response is much weaker compared with WV-specific IgG, and a higher number of doses is required to induce detectable amounts of NS1-specific IgG antibody. The weaker NS1-specific immune response compared to the WV-specific IgG response is to be expected since WV is the main component of the vaccine and NS1 is probably present in rather small amounts.

Our mouse experiments have shown that three doses representing the complete vaccination scheme are insufficient to elicit detectable levels of NS1-specific antibodies, and this could explain why there was no detection of NS1 antibodies in studies involving participants who received only the basic vaccination scheme consisting of three doses of the vaccine [7,9,10,11,12]. We previously reported that of the six serum samples obtained from TBE vaccinated individuals who had received ≤3 doses of the vaccine, five were NS1-IgG negative and one was weakly positive. However, of the 16 samples obtained from vaccinated individuals who had received >3 doses of the vaccine, 2 (12.5%) were NS1 IgG positive and 9 (56%) were weakly positive [8]. This may indeed suggest that vaccinees who received a higher number of vaccine doses could develop an anti-NS1 IgG response. However, the number of samples analyzed in our study was rather small, and it was not possible to completely exclude natural infection in these individuals, as they lived in a country highly endemic to TBE [13]. Therefore, whether people who received a higher number of doses (more than three) of FSME-Immun develop detectable NS1-specific antibodies remains unknown and will be the subject of our next study with a larger number of participants.

Experiments in mice have shown that immunization with the NS1 antigen can help protect against lethal TBEV or prolong mice survival after infection [8,14,15,16,17,18,19]. In general, the NS1 antigen is considered a promising candidate for the next generation of flavivirus vaccines [20]. Recently, however, it was found that the flavivirus NS1 immune response may also be associated with the induction of pathogenic autoreactive antibodies [20,21].

In conclusion, this and our previous study [8] suggest that any attempt to distinguish between vaccination and infection based solely on the detection of NS1 antibodies should be taken with caution.

## Data Availability

All original data are available upon request.
